# SeptoSympto: a precise image analysis of Septoria tritici blotch disease symptoms using deep learning methods on scanned images

**DOI:** 10.1186/s13007-024-01136-z

**Published:** 2024-02-01

**Authors:** Laura Mathieu, Maxime Reder, Ali Siah, Aurélie Ducasse, Camilla Langlands-Perry, Thierry C. Marcel, Jean-Benoît Morel, Cyrille Saintenac, Elsa Ballini

**Affiliations:** 1 PHIM Plant Health Institute, Univ Montpellier, INRAE, CIRAD, Institut Agro, IRD, Montpellier, France; 2 BioEcoAgro, Junia, Lille University, Liège University, UPJV, Artois University, ULCO, INRAE, Lille, France; 3 BIOGER, Paris-Saclay University, INRAE, Palaiseau, France; 4 UCA, INRAE, GDEC, Clermont-Ferrand, France; 5 PHIM Plant Health Institute, Univ Montpellier, CIRAD, INRAE, IRD, Institut Agro, Montpellier, France

**Keywords:** Septoria tritici blotch, Wheat, Image analysis, Deep learning, CNN, U-Net, YOLO

## Abstract

**Background:**

Investigations on plant-pathogen interactions require quantitative, accurate, and rapid phenotyping of crop diseases. However, visual assessment of disease symptoms is preferred over available numerical tools due to transferability challenges. These assessments are laborious, time-consuming, require expertise, and are rater dependent. More recently, deep learning has produced interesting results for evaluating plant diseases. Nevertheless, it has yet to be used to quantify the severity of Septoria tritici blotch (STB) caused by *Zymoseptoria tritici*—a frequently occurring and damaging disease on wheat crops.

**Results:**

We developed an image analysis script in Python, called SeptoSympto. This script uses deep learning models based on the U-Net and YOLO architectures to quantify necrosis and pycnidia on detached, flattened and scanned leaves of wheat seedlings. Datasets of different sizes (containing 50, 100, 200, and 300 leaves) were annotated to train Convolutional Neural Networks models. Five different datasets were tested to develop a robust tool for the accurate analysis of STB symptoms and facilitate its transferability. The results show that (i) the amount of annotated data does not influence the performances of models, (ii) the outputs of SeptoSympto are highly correlated with those of the experts, with a similar magnitude to the correlations between experts, and (iii) the accuracy of SeptoSympto allows precise and rapid quantification of necrosis and pycnidia on both durum and bread wheat leaves inoculated with different strains of the pathogen, scanned with different scanners and grown under different conditions.

**Conclusions:**

SeptoSympto takes the same amount of time as a visual assessment to evaluate STB symptoms. However, unlike visual assessments, it allows for data to be stored and evaluated by experts and non-experts in a more accurate and unbiased manner. The methods used in SeptoSympto make it a transferable, highly accurate, computationally inexpensive, easy-to-use, and adaptable tool. This study demonstrates the potential of using deep learning to assess complex plant disease symptoms such as STB.

**Supplementary Information:**

The online version contains supplementary material available at 10.1186/s13007-024-01136-z.

## Background

Septoria tritici blotch (STB), caused by the ascomycete fungus *Mycosphaerella graminicola* (Fuckel) J. Schröt. in Cohn (anamorph: *Zymoseptoria tritici* Roberge in Desmaz.), is a major and persistent threat to wheat cultivation in temperate regions [1, 2]. It is considered as the most prevalent and yield-reducing disease in Europe [3], causing up to 50% yield losses under conditions favorable for disease development [4]. The primary methods for controlling this disease involve the use of fungicides and host-resistance genes. Nearly 70% of fungicides applied in Europe are dedicated to controlling STB with a cost of up to one billion euros [5]. These strategies have limited financial sustainability and require more diversification [6]. Therefore, understanding STB symptoms is an important researched topic [7] in agro-ecological disease management [8].

STB symptoms are complex. The necrosis and the pycnidia that appear on the lesions can have diverse shapes, sizes, and colors. To exacerbate the complexity, these symptoms are highly dependent on *Z. tritici* isolates, wheat genotypes, and environmental conditions [9]. The latency period is another factor that makes STB symptoms difficult to detect. When *Z. tritici* infects wheat, leaf lesions appear only after a long symptomless period of more than ten days post-inoculation. It progressively forms necrosis that may contain pycnidia (i.e., the asexual fruiting bodies) [10]. This extended latency period makes it difficult to differentiate between the simultaneous development of disease symptoms and leaf senescence. It also makes it complicated to evaluate disease severity in an accurate manner under laboratory conditions. However, there are methods and tools that can be used to overcome these challenges.

Accurate and rapid phenotyping of STB symptoms is essential for studying this disease. The identification and quantification of pycnidia are fundamental parameters in the context of studying disease diagnosis and epidemics. Pycnidia are a distinguishing feature to detect STB in field conditions. Additionally, quantifying pycnidia can be used as an indicator of the fungus's reproductive potential and of the epidemiological development [11]. Accurate high-throughput phenotyping is necessary for carrying out genetic analyses [12], as well as for studying alternative control methods (i.e., agro-ecological approaches). These methods have low individual effects on disease in most cases. When combined, they become interesting to produce an overall effect on the disease [13]. Resistance QTLs associated with the percentage of necrosis and pycnidium production have already been identified [14]. Identifying QTLs associated with small phenotype variations is more challenging without accurate symptom detection tools. Therefore, such studies on the complex STB symptoms necessitate the development of image-based phenotyping. This type of phenotyping is increasingly used for experiments as it offers a solution to overcome the limitations of visual assessments.

Visual evaluation by raters is the most commonly used method to assess STB severity, especially in the field [15]. This type of evaluation presents challenges when quantifying the disease’s severity effectively, as described in Bock et al. [16]. Evaluation results are significantly influenced by the rater’s level of experience, making disease assessment a challenging task for young researchers. Various aspects are considered to account for rater biases, such as tools that facilitate a coordinated evaluation [17] or by proposing quantitative molecular assessments [18]. The ultimate goal is to achieve a standardized interlaboratory evaluation at the international level. The development of image analysis tools represents a means of standardizing STB phenotyping.

Among the tools available for phenotyping wheat disease symptoms, only a few methods are available to evaluate symptoms related to STB (Additional file 1, [19]). Available methods for evaluating the canopy are either based on hyperspectral remote sensing [20, 21] or on dynamic modeling and deep learning [22], aimed at differentiating this disease from other biotic and abiotic stressors. Nevertheless, none of these methods allows for an evaluation of pycnidia production. A batch processing macro in ImageJ image analysis software has been developed to automatically measure lesions and pycnidia on leaves [11, 23, 24]. Color thresholding in the RGB color space allows to measure the green leaf area and therefore lesions. The 'Find Maxima' function is used to identify pycnidia. Another method for detecting pycnidia has been developed in Python and is based on determining contours of constant brightness in the vicinity of each pycnidia [25]. More recently, a tool has been developed to detect the yellow halo present in lesion perimeters [26]. It also detects lesions based on a transformation of the original image into six color spaces, a random forest classifier for thresholding followed by a post-processed filter. Surprisingly, deep learning methods have not yet been developed to quantify STB symptoms (i.e. necrosis and pycnidia on leaves), despite the creation of tools to classify the principal foliar diseases of wheat [27, 28]. Visual analysis by experts remains the most preferred solution, although there have been efforts to create tools to detect STB symptoms.

The development of new tools remains necessary, as the tools developed so far are used by a small number of laboratories. They are time-consuming, difficult to transfer to new laboratory environment, or potentially inaccurate. Indeed, the tools developed to detect STB severity seem to be too specific to the training sets used to develop them. The development of traditional image processing techniques is labor-intensive. Each adjustment might improve results by reducing false positives for one image, but degrade results by introducing errors for another. The variability in natural samples, combined with the rigidity of predefined rules, made it a daunting task to achieve consistent and accurate results across a broad dataset. These tools are unsuitable or difficult to use with other datasets obtained under different conditions. Therefore, it is critical to develop a method for quantifying STB severity that is user-friendly, rapid, accurate, unbiased, and easily adaptable.

Several recent methods have been developed and compared for the phenotyping of plant disease symptoms. Assessing disease incidence (e.g. number of attacked plants) is relatively straightforward through visual observation. However, estimating disease severity (e.g. level of disease of individual leaf) requires complex cognition [29]. This explains the complexity of developing accurate and robust phenotyping tools, which necessarily rely on initial human assessment. Machine learning is a form of artificial intelligence capable of automatic adaptation with minimal human intervention. Deep learning is a subset of machine learning that employs highly complex neural networks. It has the potential to produce results comparable to expert assessments. Comparisons of image analysis methods for plant diseases other than STB have demonstrated that machine learning (a Support Vector Machine (SVM) classification) outperformed ImageJ (which employs thresholding and ROI mask application) in measuring the severity of cassava fire blight [30]. Furthermore, in Sujatha et al. [31], deep learning methods [i.e., Inception-v3, Visual Geometry Group (VGG-16 & VGG-19)] outperformed traditional machine learning approaches (i.e., SVM, Stochastic Gradient Descent & Random Forest) in the case of citrus disease classification. Deep learning emerges as a highly promising method for plant disease phenotyping.

Deep learning could address complex problems associated with phenotyping of STB symptoms. This method is based on an artificial neural network, which, although fundamentally different, can be linked to the human brain for data analysis and feature learning [32]. Deep learning has shown very promising results in the field of computer vision, including object detection, semantic segmentation, image classification, etc. [33]. Indeed, convolutional neural networks (CNNs) stand as the most used models for detecting plant leaf diseases. It addresses challenges inherent in traditional machine learning methods, such as symptom variations or background interference [34]. Developing a deep learning tool to quantify STB symptoms appears to be a promising method for accurately distinguishing necrosis from senescence and quantifying pycnidia. This approach allows to enhance adaptability and discernment of the complexity and variability inherent in biological images, while minimizing errors though fine-tuning model parameters. The CNNs can capture and represent hierarchical features allowing subtle variations and patterns to be discerned. Furthermore, despite the potential need for extensive training and access to labelled datasets, it provides greater accuracy and ease of adaptation compared to currently available methods. However, to our knowledge, deep learning has not yet been used for quantifying STB symptoms.

Choosing the deep learning architecture is very important to resolve problems with high performances. Deep learning architectures used for plant disease detection [35] are based on CNNs. The U-Net architecture [36] is a deep learning segmentation architecture known and widely used for its high performance [22, 37–39]. Semantic segmentation is an image analysis method that classifies each pixel of the image according to the object or class of objects to which it belongs. U-Net is simple to implement and requires a small amount of training data to be effective, which is a significant advantage for its application in biology. Models based on U-Net architectures have been shown to be effective in detecting various diseases with high performance (F1 score greater than 0.85) [37]. The choice of this architecture for necrosis detection is based on its ability to accurately identify shapes in the form of polygons. Another notable architecture is YOLOv5 (You Only Look Once version 5) architecture [40], specifically designed for real-time object detection. YOLOv5 is an object detection model that divides an image into a grid and predicts the objects present and their approximate position as rectangles. It is renowned for its speed, global optimization and end-to-end training, collectively improving detection accuracy. This architecture is considered very powerful and efficient for object detection [41]. The YOLO architecture has been applied to detect tomato diseases and pests, as it outperforms other architectures such as Faster R-CNN, Mask R-CNN and SSD in terms of accuracy and detection speed [42]. The choice of this architecture for pycnidium detection is based on its ability to perform fast and accurate detections. In this context, due to the small size and the large number of pycnidia to be identified, pycnidia are approximated by rectangles to facilitate their detection and annotation. Therefore, both U-Net and YOLOv5 architectures have the potential to enable more accurate quantification of objects such as pycnidia and polygons such as necrosis, respectively.

In this work, we tested whether deep learning techniques can accurately distinguish necrosis caused by STB from leaf senescence symptoms and precisely quantify STB necrosis and pycnidia using various datasets. We developed an image analysis script, named SeptoSympto (https://github.com/maximereder/septo-sympto). Deep learning models based on the U-Net and YOLO architectures were trained on small datasets to enable quick, quantitative and accurate phenotyping of STB symptoms obtained under controlled conditions.

## Materials and methods

### Material and image acquisition

We sourced the leaves from the third fully developed leaf and harvested it either 17 or 21 days after inoculation. To minimize sources of variability in image capture, we maintained consistent image resolution, employed fixed image backgrounds and ensured that the leaves exhibited no curvature. Each leaf was carefully fixed to an A4 sticky sheet, enclosed in a transparent pouch, and scanned. All the scanned images were captured at a resolution of 1200 dpi and saved in TIFF format to ensure compatibility with the SeptoSympto script. SeptoSympto requires the leave to be scanned horizontally. In case there was text, we used the software XnView to remove it and to rename the isolated leaves.

We selected images for the datasets with the objective of representing a maximum diversity of symptoms, while maintaining an equal distribution of each wheat line within the datasets. Different datasets were used to train and evaluate the models, as outlined in Table 1. In order to represent a maximum of possible factors influencing symptom development, these datasets originated from three laboratories, encompassed four growth conditions, featured two wheat species and included a diverse set of *Z. tritici* genotypes.

**Table 1:** Information about datasets used to develop SeptoSympto

Dataset	Usage	Leaf number	Plant species	Varieties	*Zymoseptoria* strains	Growth conditions	Scanner	Institute
1	Model training	375	*Triticum aestivum*	19	IPO9415	Greenhouse 16 h/8 h photoperiod, at 24 °C/20 °C and with 250 µmol/s/m^2^	Epson Perfection V370 Photo	1
2	Model evaluation	40	*Triticum aestivum*	19	IPO9415	Greenhouse 16 h/8 h photoperiod, at 24 °C/20 °C and with 250 µmol/s/m^2^	Epson Perfection V370 Photo	1
3	Model evaluation	50	*Triticum aestivum*	13	IPO9415	Growing chamber 16 h/8 h photoperiod, at 24 °C/20 °C and with 250 µmol/s/m^2^	Epson Perfection V370 Photo	1
4	Model evaluation	50	*Triticum turgidum*	18	P1A	Growing chamber 16 h/8 h photoperiod, at 24 °C/20 °C and with 250 µmol/s/m^2^	Epson Perfection V370 Photo	1
5	Model evaluation	115	*Triticum aestivum*	3	IPO9415	Growing chamber 16 h/8 h of photoperiod, at 21 °C/18 °C and with 400 µmol/s/m^2^	Epson Perfection V750 Pro	2
6	Model evaluation	55	*Triticum aestivum*	1	Descendants of a biparental population (Parental strains: INRA09-FS0813 & INRA09-FS0732)	Growing chamber 16 h/8 h of photoperiod at 22 °C/18 °C and with 300 µmol/s/m^2^	CanoScan 9000F MarkII	3

Six different datasets were used to create (dataset 1) or evaluate (dataset 2 to 6) the deep learning models. All the scanned images contain only wheat leaves inoculated with *Z. tritici* and were taken at 1200 dpi in TIFF format. The datasets consist of different varieties, strains, growth conditions, and scans used to evaluate the models

### Data annotation

We carried out the annotation process for individual leaf images on Roboflow. It is an online platform specially designed for data annotation to facilitate the training of computer vision models (https://roboflow.com/ or https://docs.roboflow.com/). Two independent projects, one based on semantic segmentation for necrosis, and the other on object detection for pycnidia, were created to annotate STB symptoms by an expert.

A total of 375 leaves were annotated with polygon class labels for necrosis, while 240 leaves were annotated with rectangle class labels for pycnidia. The annotation process involved zooming in on each image to its maximum extent and selectively annotating the darkest pixels, which accurately represented necrosis or pycnidia. This meticulous approach aimed to enhance the precision of annotations. Since the dimensions of necrosis or pycnidia could vary, annotations were primarily based on their distinctive colors and shapes.

Two experts performed the annotation, and subsequently a cross-validation process to ensure accuracy and consistency. Following this, the annotated data were exported in compatible formats: binary masks in PNG format for the necrosis project and coordinate tables in TXT format for the pycnidia project.

### Model training

**Fig. 1:**
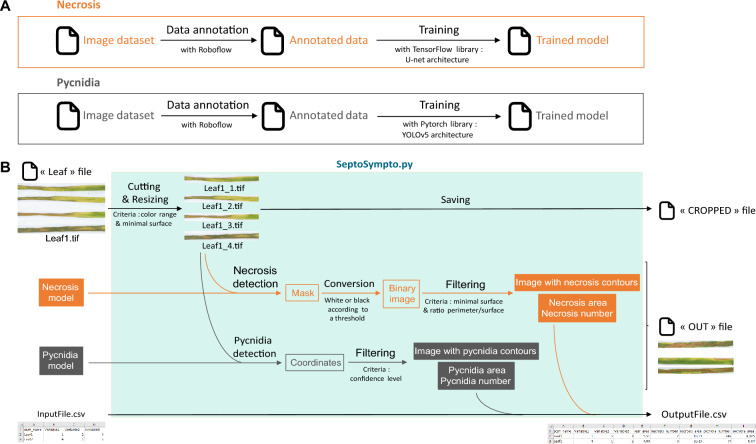
Overall workflow of model training and SeptoSympto script. **A** The images of wheat leaves inoculated with *Zymoseptoria tritici* were annotated on Roboflow in two stages: the first stage using segmentation for necrosis and the second stage using object detection for pycnidia. Necrosis annotated images were exported and a model was trained with the Tensorflow library in Python using the U-Net architecture. Pycnidia annotated images were exported and a model was trained with Pytorch library in Python using the YOLOv5 architecture. The scripts for model training are available on https://github.com/maximereder/septo-sympto. **B** To run the image analysis, a folder must contain different files as described below and the script SeptoSympto available on https://github.com/maximereder/septo-sympto. The images are stored in a file called “images_input”. A file in csv format, called “csv_input”, containing information for each image and the name of each image in the first column can be added. The models used for necrosis and pycnidia detection are stored in the “models” file. Using the input data, the script will first detect leaves according to color range and minimal surface, cut and resize each leaf. The leaves are renamed with the file name and the leaf number of the image and saved in a file called “cropped”. The second function predicts the probability of each pixel to be in necrosis class using the necrosis model, and creates a binary mask. Thresholds are applied to keep only detected ones with a minimal surface and perimeter to area ratio. The function returns the image with the necrosis contours drawn, the total area of necrosis and the necrosis number. The third function uses the pycnidia model to predict rectangles and confidence from the analyzed image, and only retains pycnidia coordinates for those with a minimal confidence level. The function returns the image with the pycnidia contours drawn, the total area of pycnidia and the pycnidia number. After running the image analysis, the output is a result csv file containing the measurements

The necrosis detection model was trained using the U-Net architecture [36], while pycnidium detection utilized the YOLO (You Only Look Once) architecture [43] (Fig. 1A). All training was conducted using Python as a programming language on a computer equipped with an Intel Core i5 processor and two T4 graphics processing units (GPU), each with 16 GB of memory.

The necrosis models used a U-Net architecture for semantic segmentation, implemented with the Tensorflow library (https://github.com/maximereder/unet). In contrast, the pycnidium models adopted the YOLOv5 architecture for object detection, implemented with the Pytorch library [40]. For both models, training was performed on datasets consisting of 50, 100, 200 or 300 annotated images and validation sets representing 20% of the largest training dataset. The input sizes of models were set to an image size of 304 × 3072 pixels.

We applied a data augmentation in the form of horizontal and vertical reversal for pycnidia, while no augmentation was employed for necrosis. During the training, the models processed 16 images per batch over 200 epochs initially. Model hyperparameters predominantly adhered to default settings. The necrosis model used Dice Loss function, while the pycnidium model employed the standard YOLO loss function, encompassing localization, classification and confidence scores. The chosen optimizer was Adam, with an initial learning rate at 0.0001 for necrosis and 0.001 for pycnidia. A patience count was employed during training to stop the process if the training metrics remain constant after 10 epochs. This count incremented with each epoch that failed to yield a superior validation loss compared to the current best. We saved the models with the best validation performance.

We saved the resulting models in.h5 format for necrosis and.pt format for pycnidia. The.h5 extension aligns with the TensorFlow library and is commonly used to save machine learning models, preserving both the segmentation model's architecture and the learned weights. Conversely, the.pt extension is the standard format for saving PyTorch models, encompassing both the model's architecture and weight parameters.

### Image analysis

The SeptoSympto script detects necrosis and pycnidia, by processing individual leaf to prepare them for analysis using deep learning models (Fig. 1B).

To initiate the image analysis, a comprehensive guide to script utilization, the SeptoSympto script and the CNN models employed for necrosis and pycnidia detection (specifically, necrosis-model-375.h5 and pycnidia-model.pt) are available at https://github.com/maximereder/septo-sympto. Additionally, several options are available such as including a CSV file containing image-related information, with each image name listed in the first column.

The SeptoSympto script encompasses three main functions. The first function within SeptoSympto script detects each leaf, employing criteria such as a minimum area and color range. Subsequently, it proceeds to cut, resize and rename the leaves. The second function focuses on predicting the probability of each pixel belonging to the necrosis class, resulting in the creation of a binary mask. This is achieved by applying thresholds based on minimum area and maximum perimeter-to-area ratio. The third function uses the pycnidia model to forecast rectangles and confidence scores from the analyzed image. It retains pycnidia coordinates that surpass a predefined confidence threshold and restricts the maximum number of pycnidia predictions per leaf. Upon completion of the image analysis, the resulting outputs encompass cropped images, images featuring pycnidia and necrosis contours and measurements containing the leaf area, necrosis area, necrosis number, pycnidia area and pycnidia number.

### Model evaluation

#### Evaluation metrics for model performance

Before implementing models in SeptoSympto script, we tested and compared several models trained with different sized datasets using metrics and expert observations. The metrics used are precision, recall and F1 for necrosis and pycnidia models:

$$Precision=\frac{TP}{TP+FP}$$

$$Recall=\frac{TP}{TP+FN}$$

$$F1 score=2\frac{Precision*Recall}{Precision+Recall}=\frac{2 TP}{2 TP+FP+FN}$$

with TP: true positive, FP: false positive and FN: false negative.

High precision denotes minimal false positive detection, signifying that the model accurately identifies the majority of true positives. High recall indicates maximum true positive detection with minimal false negatives, implying that the model effectively captures the true positives without significant omissions. The F1 score is the harmonic mean of recall and precision, with higher values indicating superior model performance.

#### Comparison with expert evaluations

We compared the results obtained from the best-performing models to expert evaluations using Spearman correlation. The Spearman correlation is a non-parametric test, making it suitable to determine the significance of the monotonic relationship between the variables tested. The Spearman’s rank correlation coefficient provides insights into the intensity of the monotonic relationship, while the p-value ascertains the significance thereof.

Experts conducted visual assessments, quantifying the area of necrosis present on the total leaf surface and the area of necrosis containing pycnidia. The process adhered to standard precision practices, rounding measurements to the nearest unit in alignment with common evaluation protocols.

## Results

### Script development and model validation

Before incorporating the final models into the SeptoSympto script, we trained a total of four models for necrosis and three for pycnidia, using different subsets of dataset 1. Three metrics were used to evaluate the models: precision, recall and F1 score.

The analysis of metrics demonstrated minimal variation across the models trained with different sizes of training datasets (Table 2), suggesting that the number of annotated leaves used for model creation could be small and this would not impact model performance. However, models N3 and P2, trained with a larger number of annotated leaves, were chosen to be integrated into the SeptoSympto script. They may allow a better representation of the diverse observable symptoms, thereby facilitating a potential improved generalization of the models for inter-laboratory use.

**Table 2:** Metrics of models for necrosis and pycnidia

A Necrosis						
Model	Leaf number in training dataset	Leaf number in validation dataset	Epochs	Precision	Recall	F1
N0	50	75	47	0.87	0.84	0.85
N1	100	75	42	0.95	0.80	0.87
N2	200	75	26	0.95	0.82	0.88
N3	300	75	20	0.95	0.85	0.90

B Pycnidia						
Model	Leaf number in training dataset	Leaf number in validation dataset	Epochs	Precision	Recall	F1
P0	50	40	199	0.54	0.27	0.36
P1	100	40	162	0.53	0.25	0.34
P2	200	40	183	0.56	0.25	0.34

The model trainings were carried out on a small number of leaves and epochs. The resulting models were evaluated using different metrics: precision, recall and F1. High precision indicates minimal false positive detection. High recall indicates maximum detection of true positives. F1 is the harmonic mean of recall and precision. **A** Four models (N0, N1, N2 & N3) for necrosis detection were trained on training datasets of different sizes, using a segmentation based deep learning architecture called U-net. **B** Three models (P0, P1 & P2) for pycnidia detection were trained on training datasets of different sizes, using an object detection based deep learning architecture called YOLOv5

The metrics allowed to select models and validate their performances. The N3 model, which is used in the SeptoSympto script for detecting necrosis, boasted a precision of 0.95, a recall of 0.85 and a F1 score of 0.90 (Table 2). This suggests that our model is adapted to detect necrosis. The loss function, which represents the cumulative errors made by the model across the training set, reached a low value after only 20 epochs (training cycles) (Additional file 2). This observation underscores the model’s ability to minimize discrepancies between expert annotations and model predictions, rendering further training unnecessary. Conversely, the P2 model, which is implemented in the SeptoSympto script for detecting pycnidia, exhibited a precision of 0.56, recall of 0.25, and F1 of 0.34 (Table 2). Given the potential of a substantial number of pycnidia detections on leaves, the metrics remained low even with 183 epochs. Detecting a large number of objects inherently presents greater difficulty in achieving high metric values. Nevertheless, the loss function curves for both the training and validation sets were decreasing (Additional file 2). This shows a reduction in errors with each iteration. The best model, based on the parameters corresponding to the lowest loss functions for both training and validation data, was therefore chosen.

**Fig. 2:**
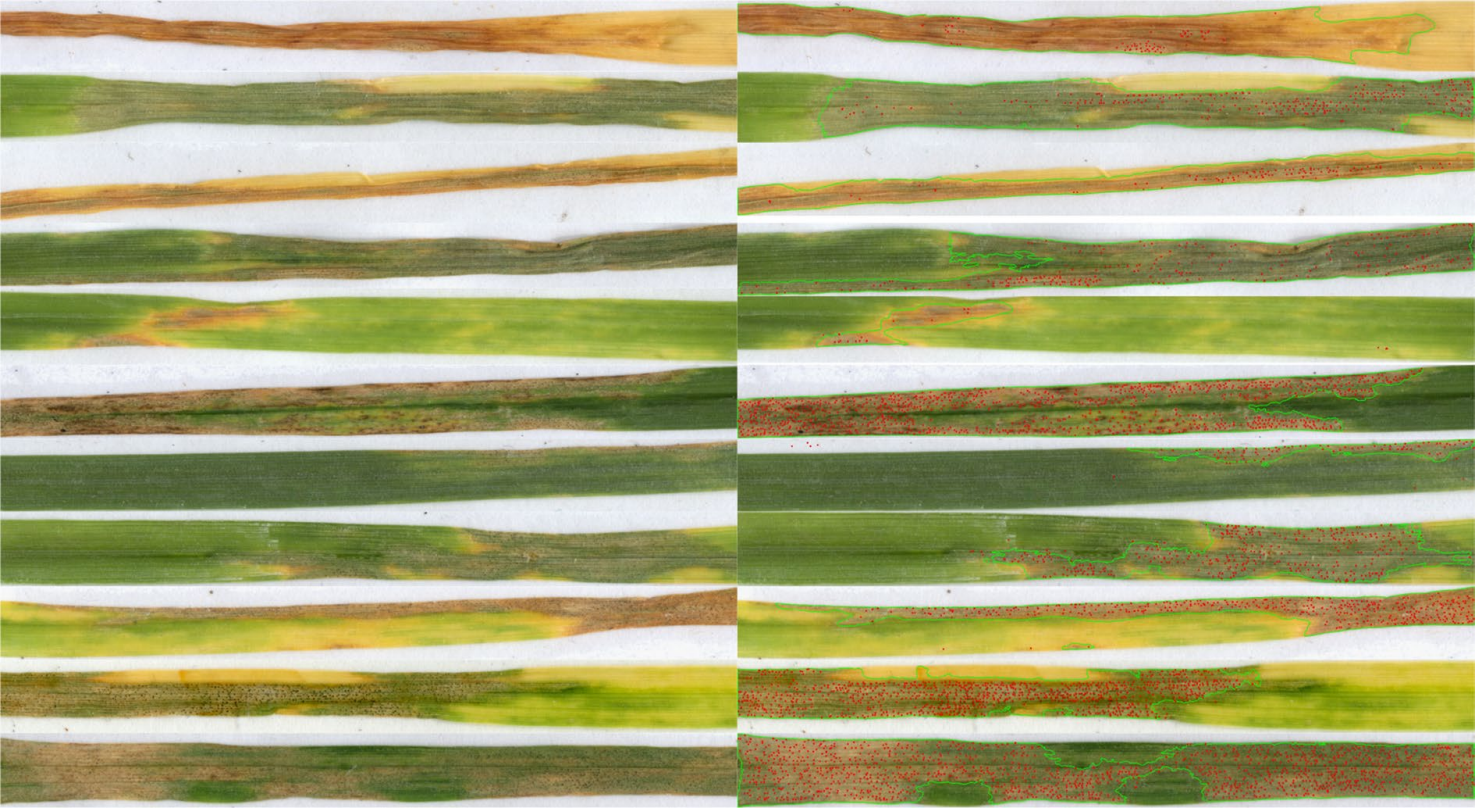
Images of *Zymoseptoria tritici* symptoms detected by SeptoSympto. The SeptoSympto image outputs after cutting, obtained in the “cropped” file, are shown on the left and the SeptoSympto image outputs after necrosis and pycnidia detection are shown on the right. The necrosis contours are in green and the pycnidia are red dots

In addition to model metrics, the observation of outputs is equally important to validate a model. Utilizing the N3 and P2 models, the observations depicted in Fig. 2 reconfirmed the models’ ability to accurately identify and quantify both pycnidia and necrosis, regardless of the quantity (absence of symptoms, necrosis without pycnidia, pycnidia density scale, necrosis size) and diversity (necrosis and pycnidia colors) of the symptoms present.

### Script evaluation

To assess the performance of the SeptoSympto script, we conducted an analysis on two independent datasets. Both were distinct from the one used for model creation. Dataset 2 contained 40 leaves, while dataset 6 encompassed 55 leaves (Table 1).

#### Evaluation of SeptoSympto using images captured under same conditions as model training

The SeptoSympto script was employed to evaluate dataset 2, alongside the assessments conducted by two independent experts. Spearman's correlation analysis was applied to examine the monotonic relationships between the SeptoSympto outputs and the expert evaluations (Table 3, Additional file 3). Our results reveled strong correlations between the script-generated outputs and the manual evaluations for both the area of necrosis present on the total leaf surface (expert 1: ρ = 0.94, p < 0.001 and expert 2: ρ = 0.75, p < 0.001) and for the area of necrosis containing pycnidia (expert 1: ρ = 0.83, p < 0.001 and expert 2: ρ = 0.80, p < 0.001). The correlation values between SeptoSympto and the experts were within the same magnitude as those obtained between the two experts themselves (necrosis: ρ = 0.74, p < 0.001 and pycnidia: ρ = 0.95, p < 0.001). This consistency was expected since deep learning models were trained on data annotated by another independent expert (expert 3).

**Table 3:** Summary of correlation results for necrosis and pycnidia detection between expert evaluations and SeptoSympto outputs across multiple assessed datasets

A Necrosis				
Dataset	Difference with the dataset 1 used for training	SeptoSympto—Expert 1	SeptoSympto—Expert 2	Expert 1 – Expert 2
2	Same conditions	p < 0.001 ρ = 0.94	p < 0.001 ρ = 0.75	p < 0.001 ρ = 0.74
3	Durum wheat	p < 0.001 ρ = 0.95	p < 0.001 ρ = 0.90	p < 0.001 ρ = 0.95
4	Different growing conditions and scanners	p < 0.001 ρ = 0.96	p < 0.001 ρ = 0.92	p < 0.001 ρ = 0.88
5		p < 0.001 ρ = 0.83		
6		p < 0.001 ρ = 0.76		

B Pycnidia				
Dataset	Difference with the dataset 1 used for training	SeptoSympto—Expert 1	SeptoSympto—Expert 2	Expert 1–Expert 2
2	Same conditions	p < 0.001 ρ = 0.83	p < 0.001 ρ = 0.80	p < 0.001 ρ = 0.95
3	Durum wheat	p < 0.001 ρ = 0.58	p < 0.001 ρ = 0.81	p < 0.001 ρ = 0.80
4	Different growing conditions and scanners	p < 0.001 ρ = 0.69	p < 0.001 ρ = 0.71	p < 0.001 ρ = 0.89
5		p < 0.001 ρ = 0.81		
6		p < 0.001 ρ = 0.94		

Spearman correlations were used to compare expert assessments and SeptoSympto measurements of necrosis and pycnidia. The Spearman's rank correlation coefficient (ρ) and the p-values (p) are given for each comparison. The datasets were obtained from various conditions as shown in Table 1

#### Comparison of SeptoSympto with ImageJ macro.

**Fig. 3:**
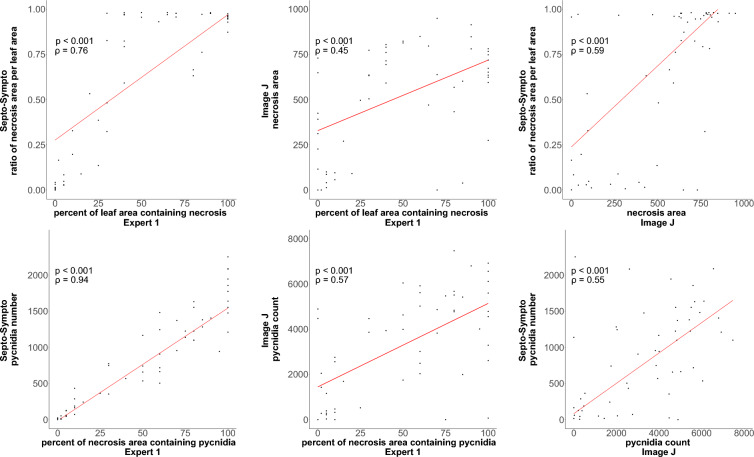
Correlations between expert ratings, Image J and SeptoSympto outputs for necrosis and pycnidia detection in dataset 6. Comparative analysis of expert assessments, Image J and SeptoSympto for the measurements of necrosis and pycnidia on 55 bread wheat leaves scanned with the CanoScan 9000F MarkII scanner was conducted using Spearman correlations. Dataset 6 was obtained under different growing conditions, with different *Z. tritici* isolates and a different variety from the dataset used for the model trainings

To compare the different tools available for STB phenotyping, we submitted dataset 6 to the tool developed by Steward & McDonald 2014 and Stewart et *al*. 2016 [23, 24], to the SeptoSympto script and to a visual evaluation (Fig. 3). Correlations between the two tools were modest (pycnidia number: ρ = 0.55, p < 0.001 and necrosis area: ρ = 0.59, p < 0.001). However, SeptoSympto exhibited higher correlations with expert 1 (necrosis: ρ = 0.76, p < 0.001 and pycnidia: ρ = 0.94, p < 0.001) compared to those observed between ImageJ and expert 1 (necrosis: ρ = 0.45, p < 0.001 and pycnidia: ρ = 0.57, p < 0.001). It should be noted that expert 1 is independent from the expert 3, who developed SeptoSympto. Therefore, this observation underscores that SeptoSympto surpasses the previous tool [23], as a more accurate tool to quantify STB symptoms.

### Script transfer

A major problem in phenotyping based on machine learning techniques is the risk of overfitting, where the model becomes too specific to the training set. However, the analyses presented above demonstrated that our script does not exhibit any overfitting. It effectively detects necrosis and pycnidia in datasets that were not part of the model training (Table 3). It is worth noting that dataset 2 was obtained under the same conditions (same varieties, strain, growing conditions and scanner) as the training set. To ensure the tool’s applicability across diverse conditions, we investigated whether SeptoSympto performed equally well on datasets from different conditions, including varying species, varieties, strains, growing conditions, and scanners, compared to the dataset used to create the models.

#### Evaluation of SeptoSympto using images obtained from different growing conditions

We initiated the assessment by evaluating dataset 3, obtained under similar conditions (varieties, strain and scanner) but with variations in growing conditions. SeptoSympto exhibited high correlations with expert assessments for both necrosis (expert 1: ρ = 0.95, p < 0.001 and expert 2: ρ = 0.90, p < 0.001) and pycnidia (expert 1: ρ = 0.58, p < 0.001 and expert 2: ρ = 0.81, p < 0.001) (Additional file 4). The correlation between the experts was also high (necrosis: ρ = 0.95, p < 0.001 and pycnidia: ρ = 0.80, p < 0.001), similar to the results obtained with dataset 2. These findings suggest that SeptoSympto can effectively adapt to datasets from different growing conditions.

#### SeptoSympto evaluation using durum wheat images

To further evaluate its performance, we tested SeptoSympto capability on dataset 4 encompassing leaves of another species: durum wheat inoculated with a different strain but grown under conditions similar to those in dataset 3 and scanned using the same scanner. The correlations between SeptoSympto results and expert manual scoring remained high (Additional file 5A) for necrosis (expert 1: ρ = 0.96, p < 0.001 and expert 2: ρ = 0.92, p < 0.001; correlation between the experts: ρ = 0.88, p < 0.001) and pycnidia (expert 1: ρ = 0.69, p < 0.001 and expert 2: ρ = 0.71, p < 0.001; correlation between the experts: ρ = 0.89, p < 0.001). This indicates that SeptoSympto performs effectively across datasets from both bread and durum wheat species, even with different strains of *Z. tritici*.

#### SeptoSympto evaluation using images from different growing conditions and scanners

Furthermore, we assessed whether the script performance was sensitive to image capture from different laboratories. Dataset 5 was composed of 115 leaves from three varieties already present in datasets 1, 2 and 3, inoculated with the same strain as these three datasets, but grown under distinct conditions and scanned using a different scanner. The correlations obtained between SeptoSympto and expert 1 results remained high (necrosis: ρ = 0.83, p < 0.001 and pycnidia: ρ = 0.81, p < 0.001) (Additional file 5B). Lastly, dataset 6 comprised 55 leaves from the same variety, inoculated with different isolates, and imaged with a different scanner while maintaining the same resolution. The correlations between SeptoSympto and expert 1 did not decrease (necrosis: ρ = 0.76, p < 0.001 and pycnidia: ρ = 0.94, p < 0.001) (Fig. 3), indicating that SeptoSympto can effectively analyze images from different scanners. These results emphasize that SeptoSympto is a robust tool for accurate analysis of STB symptoms under different experimental conditions.

## Discussion

With the emergence of artificial intelligence, high-throughput phenotyping is growing exponentially, encompassing not only visible spectrum images but also other spectral ranges. This approach allows the rapid, easy and reproducible acquisition of high-quality phenotyping data. However, the phenotyping of STB symptoms in wheat is still predominantly reliant on visual assessment, a labor-intensive and time-consuming process that requires expertise [44]. To address this challenge, we have developed an image analysis script using deep-learning techniques. It allows precise phenotyping of necrosis and pycnidia caused by STB with pre-trained Convolutional Neural Networks. CNN architectures lessen the burden of image annotation and enable more accurate quantification of STB symptoms from scanned leaves of wheat seedlings, notably for pycnidium detection. Our script, named SeptoSympto, employs two models trained on small datasets: one model trained with the U-Net architecture for necrosis detection via semantic segmentation, and the other one trained with the YOLO v5 architecture that distinguishes pycnidia via object detection. High correlations between expert assessments and SeptoSympto outputs were obtained with 6 different datasets used to evaluate the script. These datasets are composed of images obtained with 3 different scanners at 1200 dpi and including 38 varieties of bread wheat or durum wheat, inoculated with different *Z. tritici* strains, and grown under diverse conditions.

SeptoSympto currently stands as the sole available laboratory tool available for the phenotyping of STB, employing innovative methodologies like CNNs (Additional file 1). A dataset obtained by a different laboratory than the ones that developed SeptoSympto and the ImageJ macro [23] was evaluated to compare the two tools. It has become evident that the use of state-of-the-art CNN architectures improves the reliability of STB detection. Additionally, a recent field study on STB phenotyping [22] employed the U-Net architecture, the same one implemented in SeptoSympto for necrosis quantification, demonstrating its effectiveness in field disease detection. The use of deep learning models to quantify STB symptoms improved the accuracy compared with other image-based tools. The SeptoSympto tool also offers advantages over visual assessment of STB symptoms.

Criteria such as analysis time, data storage, accuracy and usability are important to take into account when comparing a new tool with the most commonly used method: visual assessment. To obtain models, the time required to annotate the images (1 h for 10 leaves) and to train the model (1 to 3 h depending on the number of leaves) remains low for deep learning models. Smaller amounts of data are annotated for training. In term of image analysis, additional time is needed for image acquisition compared to visual evaluation, as leaves have to be collected, pasted and scanned (60' for 80 leaves). However, the execution time of the script for cropping the images and detecting necrosis and pycnidia (1′45″ for 10 leaves) is nearly equivalent to visual evaluation time (2′22″ for 10 leaves). With a visual evaluation, we obtain quantitative data stored in a table. This requires minimal storage space, but it forfeits access to raw data (the leaf observation). In addition, visual evaluation requires expertise and makes it possible to obtain only the areas of leaves containing necrosis or of necrosis containing pycnidia, but not the number of pycnidia. Conversely, SeptoSympto retains scanned leaf images based on the chosen script outputs. Moreover, this image analysis tool yields more precise data. The number of pycnidia may be a better proxy for assessing sporulation capacity compared to visually assessed leaf area containing pycnidia. A better detection of this pycnidia number could allow a better evaluation of the aggressiveness of STB strains [45]. Detection with SeptoSympto also avoids differences of notations between experts. Variability in estimation regarding the leaf area covered by pycnidia can be observed among experts, depending on pycnidia density or the size within this area. Here, the raters possess over a decade of expertise on STB. Using the SeptoSympto tool, non-experts can also accurately study the STB severity. This tool is intended to be comparable in accuracy to expert assessments, while also providing additional advantages such as user-friendliness for both experts and non-experts, data storage or rapid analysis, thanks to models adapted to the problems.

Developing deep learning models requires controlling source variability, as well as methodological and dataset issues [32]. The datasets used to train necrosis and pycnidium detection models are composed of images obtained from a standardized acquisition and encompass a small number of annotated data. The models can therefore be easily recreated. When the training dataset is small, it reveals the effectiveness of the chosen detection method [46]. Therefore, the architectures used were carefully chosen to best suit our problems. Under- and over-constraining the model may lead to suboptimal performances. Hence, achieving optimal performance relies on having an adequate amount of training data. In the case of SeptoSympto, varying training dataset sizes did not result in performance improvements, indicating that the optimal training dataset size had been reached. Furthermore, we have developed a tool that is as frugal as possible [47], in order to build the most accurate models possible with minimal resource consumption. As a result, SeptoSympto is an end-to-end script coded in Python using deep learning models. It executes quickly and offers several options for selecting input and output files. The methods used to develop the script were chosen to be not only the most powerful but also the most frugal and easy to use. Models are trained on small datasets and can be trained on computers with different processors and graphic cards to facilitate the training of new models. All this allows to easily annotate images and train necrosis and pycnidium detection models.

Key issues in deploying new phenotyping methods involve their transferability between laboratories and acceptance by the research community. The SeptoSympto tool was developed with the aim of being efficient across a broad spectrum of datasets scanned at 1200 dpi, as well as easily adaptable to other datasets. To avoid a common problem with deep learning models: overfitting [46], a kind of cross-validation to validate the models implemented in SeptoSympto was performed on several datasets with expert notations. The model metrics may appear modest, especially for pycnidia. However, high correlations between script outputs and expert ratings, unaffected by dataset changes, support the robustness of our models as well as the absence of overfitting. Therefore, developing SeptoSympto allowed to create a precise tool for STB phenotyping while facilitating its adaptation and transferability, in order to allow standardization of inter-laboratory assessments of STB symptoms.

Depending on the applications -plot management, rate phenotyping or epidemiological surveys-, tools based on image analysis to detect plant diseases can be developed at various detection scales: at the level of the leaf, the plant, or the cover in order to quantify, identify or detect early the disease. In the case of SeptoSympto, it is a leaf phenotyping tool allowing the accurate quantification of STB severity for necrosis and pycnidia. While our current models for SeptoSympto offer precise detection and script transferability, we could create an open-access database with annotated data to train the models on larger datasets, encompassing leaves scanned by numerous laboratories, to allow the use of a single script at the international level which is as robust as possible. In addition, the script could be adapted to detect STB symptoms in the field, which are very different from the symptoms observed under control conditions and where other diseases could be observed on the same leaves. This adaptation to the field data would be easily achieved by using a portable scanner and training new models.

## Conclusion

Septoria tritici blotch (STB) is an extensively studied disease due to its significant and persistent impact on wheat cultivation. A major challenge in this context is the development of an automated phenotyping tool capable of accurately and efficiently analyzing symptoms from scanned images while maintaining transferability. To address this challenge, we developed the SeptoSympto tool. It is a script coded in Python that uses deep learning models to detect STB symptoms. The models were trained on a relatively small dataset to facilitate the tool’s transferability. Necrosis and pycnidia were quantified using the U-Net and YOLOv5 architectures. The primary objective behind this script development was to develop an easily adaptable and common tool between laboratories to assess STB symptoms. Therefore, we have developed a tool capable of accurate and rapid quantification of necrosis and pycnidia on durum and bread wheat leaves inoculated with different strains, grown in different conditions and scanned with different scanners.

### Supplementary Information

**Additional file 1.** Comparison of image analysis methods for detection and quantification of STB. Comprehensive overview of published tools employed for STB phenotyping and their key differentiating factors. These factors include the growth conditions of captured images, the approach for image acquisition, the techniques for STB detection and quantification, as well as the capacity to detect or quantify necrosis and pycnidia.

**Additional file 2.** Loss curves of model training by epochs for necrosis and pycnidia. (A) Loss curves by epochs obtained for training the N3 necrosis model, with 300 leaves in the training dataset (red dots) and 75 leaves in the validation dataset (blue dots). (B) Loss curves by epochs obtained for training the P2 pycnidia model, with 200 leaves in the training dataset (red dots) and 40 leaves in the validation dataset (blue dots).

**Additional file 3.** Correlations between expert ratings and SeptoSympto results for necrosis and pycnidia detection in test dataset 2. Comparative analysis of expert assessments and SeptoSympto for the measurements of necrosis and pycnidia on 40 bread wheat leaves scanned with the Epson Perfection V370 Photo scanner was conducted using Spearman correlations. Dataset 2 was obtained under the same growth conditions, with the same Z. tritici strain and varieties as the dataset used for the model trainings.

**Additional file 4.** Correlations between expert ratings and SeptoSympto results for necrosis and pycnidia detection in dataset 3. Comparative analysis of expert assessments and SeptoSympto for the measurements of necrosis and pycnidia on 50 bread wheat leaves scanned with the Epson Perfection V370 Photo scanner was conducted using Spearman correlations. Dataset 3 was obtained under different growing conditions, with the same Z. tritici strain and 13 same varieties from the dataset used for the model trainings.

**Additional file 5.** Correlations between expert ratings and SeptoSympto results for necrosis and pycnidia detection in datasets 4 (**A**) and 5 (**B**). **A** Comparative analysis of expert assessments and SeptoSympto for the measurements of necrosis and pycnidia on 50 durum wheat leaves scanned with the Epson Perfection V370 Photo scanner was conducted using Spearman correlations. Dataset 4 was obtained under different growing conditions, a different Z. tritici strain and 18 different varieties from the dataset used for the model trainings. **B** Comparative analysis of expert assessments and SeptoSympto for the measurements of necrosis and pycnidia on 115 bread wheat leaves scanned with the Epson Perfection V750 Pro scanner was conducted using Spearman correlations. Dataset 5 was obtained under different growing conditions, with the same Z. tritici strain and 3 same varieties from the dataset used for the model trainings.

## Data Availability

The datasets used in this study are available from the corresponding author on reasonable request.
